# Nuclear poly(ADP-ribose) activity is a therapeutic target in amyotrophic lateral sclerosis

**DOI:** 10.1186/s40478-018-0586-1

**Published:** 2018-08-29

**Authors:** L. McGurk, J. Mojsilovic-Petrovic, V. M. Van Deerlin, J. Shorter, R. G. Kalb, V. M. Lee, J. Q. Trojanowski, E. B. Lee, N. M. Bonini

**Affiliations:** 1 Department of Biology, University of Pennsylvania, Philadelphia, PA 19104 USA; 2 Department of Neurology, Children’s Hospital of Philadelphia, Joseph Stokes Jr. Research Institute, Philadelphia, PA 19104 USA; 3 Present address: Les Turner ALS Center at Northwestern Medicine, Feinberg School of Medicine, Northwestern University, Chicago, IL 60611 USA; 4 Department of Pathology and Laboratory Medicine, Perelman School of Medicine, Philadelphia, PA 19104 USA; 5 Department of Biochemistry and Biophysics, Perelman School of Medicine at the University of Pennsylvania, Philadelphia, PA 19104 USA; 6 Translational Neuropathology Research Laboratory, 605B Stellar Chance Laboratories, 422 Curie Blvd, Philadelphia, PA 19104 USA

**Keywords:** ABT-888/Veliparib, Parp, Poly(ADP-ribose), PAR, PARylation, Motor neuron disease, primary neuron, TDP-43

## Abstract

Amyotrophic lateral sclerosis (ALS) is a devastating and fatal motor neuron disease. Diagnosis typically occurs in the fifth decade of life and the disease progresses rapidly leading to death within ~ 2–5 years of symptomatic onset. There is no cure, and the few available treatments offer only a modest extension in patient survival. A protein central to ALS is the nuclear RNA/DNA-binding protein, TDP-43. In > 95% of ALS patients, TDP-43 is cleared from the nucleus and forms phosphorylated protein aggregates in the cytoplasm of affected neurons and glia. We recently defined that poly(ADP-ribose) (PAR) activity regulates TDP-43-associated toxicity. PAR is a posttranslational modification that is attached to target proteins by PAR polymerases (PARPs). PARP-1 and PARP-2 are the major enzymes that are active in the nucleus. Here, we uncovered that the motor neurons of the ALS spinal cord were associated with elevated nuclear PAR, suggesting elevated PARP activity. Veliparib, a small-molecule inhibitor of nuclear PARP-1/2, mitigated the formation of cytoplasmic TDP-43 aggregates in mammalian cells. In primary spinal-cord cultures from rat, Veliparib also inhibited TDP-43-associated neuronal death. These studies uncover that PAR activity is misregulated in the ALS spinal cord, and a small-molecular inhibitor of PARP-1/2 activity may have therapeutic potential in the treatment of ALS and related disorders associated with abnormal TDP-43 homeostasis.

## Introduction

Amyotrophic lateral sclerosis (ALS) is a fatal neurodegenerative disease where the degeneration of upper and lower motor neurons leads to muscle atrophy, paralysis and death typically within ~ 2–5 years of disease onset [47]. In > 95% of ALS patients, the normally nuclear protein TDP-43 redistributes to the cytoplasm and forms phosphorylated aggregates in affected neurons and glia [45, 69, 84, 85]. The treatment options for ALS are bleak, most are palliative and address the well-being and comfort of the patient [38, 44, 82, 91]. The first FDA-approved drug was riluzole, an anti-glutamatergic that provides a ~ 2–3-month extension in patient survival [88, 95]. In the 20 years since, a gamut of treatments has been clinically tested, but most have failed to demonstrate therapeutic efficacy [9, 38, 88]. In 2017, the second FDA approval was granted to edaravone, an anti-oxidant which, when administered with riluzole, modestly reduces neurological decline in the early stages of disease [26, 39, 40, 92, 97]. Thus, uncovering molecular pathways that contribute to the decline and loss of motor neurons in ALS is imperative for the development and testing of new treatments.

Although the exact cause of ALS remains largely unknown, genetic factors contribute to ~ 5–10% of cases [65, 107]. Familial genes include *SOD1*, *C9orf72*, *ATXN2* and *TARDBP* [28, 29, 89, 90, 104]. Several of the proteins mutated in ALS, including TDP-43 and Ataxin-2, are components of cytoplasmic stress granules [64], which are membraneless organelles that are comprised of translationally-arrested mRNA and associated proteins [4, 53]. In the ALS spinal cord, several stress-granule proteins, such as TIA-1, eIF3, and PABPC-1, co-aggregate with phosphorylated TDP-43 inclusions [10, 66, 74]. Furthermore, manipulation of proteins that regulate the stress response is beneficial in animal and cellular models of ALS [8, 29, 35, 55, 58, 86, 98, 117]. Despite evidence implicating stress pathways in ALS, it is unclear whether they are cause or consequence of the disease process.

We identified Tankyrase, a poly(ADP-ribose) polymerase, or PARP, as a potent regulator of disease-associated features of TDP-43 in *Drosophila* and mammalian cell models of ALS [73]. PARPs are enzymes that catabolize NAD^+^ to sequentially add ADP-ribose subunits onto target proteins, generating polymers of poly(ADP-ribose) (PAR) [37]. PAR activity is often stress responsive and can serve as an upstream signaling molecule [41, 63, 68]. In mammals, the PARP superfamily consists of 17 enzymes, with the most abundant and well characterized being PARP-1 [67, 99]. In the nucleus, PARP-1 and PARP-2 regulate DNA damage, gene expression, and cell survival [18, 34, 41, 48, 67]. Here, we report that PAR levels are elevated in the nuclei of motor neurons in the spinal cord of ALS patients, and that a PARP-1/2 inhibitor is therapeutic in a rodent spinal-cord cellular model of TDP-43-associated toxicity. These findings implicate an alteration in PAR activity in ALS, and suggest that PARP-1/2 inhibitors, which are in use for cancer treatment, might be repurposed for TDP-43-associated disorders.

## Materials and methods

### Clinical data and patient consent

Patient tissue was obtained from the Center for Neurodegenerative Disease Research (CNDR) Brain Bank at the University of Pennsylvania, brief details are provided in Tables 1 and 2. Patients were selected on the basis of having phosphorylated TDP-43 in motor neurons in the spinal cord. All patients pre-consented for autopsy as well as at time of death. Consent for autopsy was re-obtained from the next-of-kin in accordance with institutional review board guidelines of the University of Pennsylvania. The University of Pennsylvania Institutional Review Board reviewed and confirmed that the CNDR Neurodegenerative Disease Autopsy Brain Bank protocols meet the criteria for human-subjects research.

**Table 1:** Patients with no known neurological disease

#	Diagnosis	Sex	Age at Death (yr)	PMI (hr)	Brain weight (g)	ALS stage	Braak stage	Thal phase	CERAD	LBD
1	normal	M	47	12	1383	0	I/II	0	0	no
2	normal	M	70	10.5	1388	0	I/II	1	0	no
3	normal	F	72	7	1406	0	I/II	0	0	no
4	normal	F	65	19	1207	0	0	1	0	no
5	normal	F	56	12	1416	0	I/II	n/a	0	no
6	normal	M	61	6	1369	0	0	1	0	no
7	normal	M	55	11.5	1448	0	0	n/a	0	no
8	normal	F	59	13	1166	0	0	n/a	0	no
9	normal	M	68	21	1330	0	I/II	0	0	no
10	normal	M	47	11	1333	0	I/II	n/a	A	no
11	normal	M	72	13.5	1320	0	I/II	3	A	no
12	normal	F	46	12	1228	0	0	n/a	0	no
13	normal	F	65	22	1206	0	I/II	1	0	no
14	normal	M	67	15	1545	0	I/II	2	A	no
15	normal	F	68	15	1151	0	I/II	0	0	no
16	normal	M	70	36	1755	0	0	0	0	no

Abbreviations: #: case number, *Normal* diagnosed neurologically normal, *F* female, *M* male, *PMI* postmortem interval, *ALS stage* stages 0–4 semiquantitatively assessed according to [14, 15]. *Braak stage* neurofibrillary tangle deposition according to [12, 13]. *Thal phase* amyloid deposition according to [108]. *CERAD* neuritic plaque deposition according to [76, 80]. *LBD* Lewy Body disease according to [75]. *n/a* data not available. *no* no LBD

**Table 2:** Details of patients diagnosed with ALS-related neurological disease

#	Diagnosis	Sex	Age of Onset (yr)	Age at Death (yr)	Disease Duration (yr)	Mutation Status	PMI (hr)	Brain weight (g)	ALS Stage	Braak stage	Thal phase	CERAD	LBD
17	ALS	M	41	42	1	–	8	1554	2	I/II	n/a	0	no
18	ALS	M	71	76	5	–	23	1297	1	I/II	n/a	A	no
19	ALS	M	50	53	3	–	24	1422	2	0	n/a	0	no
20	ALS-D	F	50	51	1	–	4	1203	4	I/II	n/a	0	no
21	ALS	M	43	46	3	–	5	1427	3	I/II	n/a	0	no
22	ALS	F	79	81	2	–	10	1215	4	III/IV	n/a	0	no
23	ALS	M	64	66	2	–	14	1427	2	I/II	n/a	0	no
24	ALS	M	76	85	9	–	9	1041	1	*V*/VI	n/a	C	diffuse neocortical
25	ALS	F	73	75	2	–	8	1405	4	I/II	n/a	A	no
26	ALS-D	F	57	59	2	–	18	1125	4	I/II	n/a	0	no
27	ALS/PLS	M	54	74	20	–	4	1169	1	I/II	n/a	0	no
28	ALS	M	69	70	1	–	4	1135	1	0	0	0	no
29	ALS	F	63	67	4	–	10	1384	2	0	0	0	no
30	ALS	F	43	50	7	–	n/a	1237	2	0	0	0	no
31	ALS	F	n/a	48	n/a	*ATXN2 (22/32)*	5	1374	3	0	n/a	0	no
32	ALS-D	M	n/a	78	n/a	*ATXN2 (22/27)*	6	1300	4	III/IV	n/a	B	transitional
33	ALS	F	64	67	3	*ATXN2 (20/31)*	19	1229	3	I/II	n/a	0	no
34	ALS	M	63	65	2	*ATXN2 (22/29)*	7	1395	4	III/IV	n/a	0	no
35	ALS	F	54	56	2	*ATXN2 (*22/27)	10	1426	1	I/II	n/a	0	no
36	ALS	M	52	54	2	*C9orf72*	4	1536	3	0	n/a	0	no
37	FTD	F	47	54	7	*C9orf72*	12	813	4	III/IV	n/a	0	no
38	ALS-D	M	55	57	2	*C9orf72*	9	1200	4	III/IV	n/a	B	no
39	ALS-D	M	54	57	3	*C9orf72*	15	1244	n/a	I/II	n/a	0	no
40	ALS-D	F	67	69	2	*C9orf72*	21	1079	4	III/IV	n/a	B	no
41	ALS-D	M	61	62	1	*C9orf72*	30	1240	4	I/II	n/a	0	no
42	ALS-D	M	46	48	2	*C9orf72*	13	1309	4	I/II	n/a	0	no
43	ALS	M	70	71	1	*C9orf72*	18	1221	2	V/VI	2	B	no

Abbreviations: #: case number. -: no known mutation in *TARDBP*, *UBQLN2*, *ATXN2*, and *C9orf72*. *ATXN2* refers to an intermediate CAG-trinucleotide expansion in *ATXN2* (pathologic repeat length is indicated in brackets). *C9orf72* refers to a GGGGCC-hexanucleotide repeat expansion. *ALS-D* ALS with dementia, *FTD* frontotemporal degeneration, *PLS* primary lateral sclerosis. *F* female, *M* male. *PMI* postmortem interval. *ALS stage* stages 0–4 semiquantitatively assessed according to [14, 15]. *Braak stage* neurofibrillary tangle deposition according to [12, 13]. *Thal phase* amyloid deposition according to [108]. *CERAD* neuritic plaque deposition according to [76, 80]. *LBD* Lewy Body disease according to [75]. *n/a* data not available. *no* no LBD

### Immunohistochemistry

Tissue was examined by routine neuropathologic diagnostic methods, as described [36, 83, 85, 110]. Briefly, spinal-cord regions were fixed in 10% neutral buffered formalin and 6 μm thick sections were cut from paraffin-embedded tissue. After dewaxing and rehydration endogenous peroxidases were quenched in 30% H_2_O_2_ made up in methanol (30 min) and washed in running tap water (10 min). For antibodies requiring antigen retrieval (only anti-phosphorylated TDP-43) slides were incubated in a citrate based antigen retrieval (pH 6) buffer (Vector labs #H3300) (15 min at 99 °C) in an EZ-retriever microwave (BioGenex). Slides and solution were placed in a cool tray and left to cool to room temperature (~ 20 min). Slides were washed in 0.1 M Tris pH 7.6 and blocked in 0.1 M Tris pH 7.6 with 2% FBS. Primary antibodies, in 0.1 M Tris pH 7.6 with 2% FBS, were applied overnight at 4 °C. Sections were washed in 0.1 M Tris pH 7.6, blocked in Tris pH 7.6 with 2% FBS, and incubated with biotinylated IgG from mouse (1 in 1000, Vector labs #BA-2000) or rat (1 in 1000, Vector labs #BA-9401) for 1 h at room temperature. Slides were washed in 0.1 M Tris pH 7.6 and then 0.1 M Tris pH 7.6 with 2% FBS and incubated with an avidin-conjugated horseradish peroxidase (Vectastain ABC kit, #PK-6100) made up in Tris pH 7.6 with 2% FBS (1 h at room temperature). Slides were washed in Tris pH 7.6 and developed with Diaminiobenzidine (DAB) solution (Vector labs, SK-4105) for 8 min at room temperature. Slides were counterstained with Harris hematoxylin (30 s), washed in running tap water (10 min) dehydrated, cleared in xylene and mounted in cytoseal XYL (ThermoFisher, #8312–4). All Tris based washes were 5 min. Primary antibodies used were rat anti-phosphorylated (pS409/410) TDP-43 monoclonal antibody (1 in 500, [83]) and mouse anti-PAR, BSA free (1 in 500, Tulip Biolabs, #1020 N). Note, antigen retrieval and cooling steps were omitted for anti-PAR labelling. The anti-PAR antibody was first optimized by a serial dilution test (from 1 in 400 to 1 in 25, 000). No signal was detected at 1 in 25, 000 indicating that the secondary antibody was not contributing to the observed signal at higher primary concentrations. Dilution tests were performed on spinal cord tissue from 5 normal cases and 4 ALS cases.

Slides were coded and blinded and quantified by two researchers independently. For nuclear PAR scoring, 1–5 sections from every case were quantified and all the alpha motor neurons present in the anterior horns of each of section were scored for whether the nucleus was present and, if so, whether the nucleus stained for PAR. If all motor neurons with a nucleus were negative for PAR the score was 0. If 1 or more motor neurons with nuclei visible in the section were present and stained for nuclear PAR: a score of + was given if nuclear PAR was present in 1 motor neuron and ++ if more than 1 motor neuron stained for nuclear PAR. To determine the number of alpha motor neurons in ALS and normal spinal cord, motor neurons from one anterior horn from each case were counted. In the ALS anterior horn, there were 13.7±1.4 (SEM) alpha motor neurons with 4.0±0.4 (SEM) nuclei exposed. In the normal anterior horn, there were 19.5±1.4 (SEM) alpha motor neurons with 5.6±0.6 (SEM) nuclei exposed.

### Immunofluorescence and cell culture

Human TDP-43-YFP cloned into pcDNA3.2 is described [29]. Standard cell culture and immunofluorescence techniques were used as described [73]. Briefly, COS-7 cells were maintained in Dulbecco’s modified Eagle’s medium (DMEM) containing high glucose and L-glutamine and sodium bicarbonate (Sigma-Aldrich, #D5796. 10% fetal bovine serum (Sigma-Aldrich, #F6178) and 1% penicillin-streptomycin (ThermoFisher, #15140122) at 37 °C with 5% CO_2_. For immunofluorescence cells were grown on glass coverslips coated with poly-L-lysine (NeuVitro, #H-12-1.5-pll) and transfected with Lipofectamine LTX and PLUS reagent (ThermoFisher, #15338100) in DMEM with 10% fetal bovine serum and no antibiotics. The transfection reaction was not removed and experiments were performed 21 h later. Veliparib (ABT-888, Selleckchem, # S1004) experiments were performed by supplementing the media with the inhibitor, cells were pre-treated with Veliparib or DMSO for 90 min prior to stress. Cells were then incubated for 30 min with media supplemented with 0.25 mM sodium arsenite and DMSO or Veliparib at the indicated concentration. Cells were fixed for 15 min in 4% paraformaldehyde, permeabilized in PEM buffer (100 mM PIPES, 1 mM MgCl_2_ and 10 mM EGTA pH 6.8) supplemented with 0.1% triton X100 and then blocked in 10% normal donkey serum (Sigma-Aldrich). The primary antibody used was anti-mouse TIAR (1 in 500; BD Biosciences #5137) and the secondary antibody was Alexa-Fluor-594 (1 in 500; ThermoFisher, # A-21203). Coverslips were mounted in Prolong Diamond (ThermoFisher, # P36965). 4–5 independent images were captured at 20X magnification and the percentage of cells with cytoplasmic YFP-positive foci or TIAR-labelled stress granules were quantified. Approximately five to ten images were captured per experiment and each experiment was performed at least three independent times. Statistics were carried out using Graphpad 6 software.

### Rat motor neuron cultures

Mixed spinal cord cultures were prepared from rat and transfected with virus following previously established protocols [77, 78]. The titer of herpes simplex virus routinely used in our studies was 3-5 × 10^7^ plaque forming units (pfu)/ml [78]. The primary neuron cultures were infected 14 days in vitro (DIV) with herpes simplex virus (HSV) expressing either TDP-43 or LacZ. The inhibitor Veliparib, also called ABT-888 (Selleckchem, # S1004), or DMSO was added to the cell-culture medium at the indicated concentration at the time of infection. Media was changed 3-days post infection and cells were fixed and processed for immunofluorescence on day 5 of infection. Mouse anti-neurofilament-H, NF-H (1 in 1000, Biolegend #801703) and mouse AlexaFluor 488 (1 in 500, ThermoFisher, # A-21203) were used to identify neurons. Five images (10X magnification) were captured from each condition and remaining neuronal cell bodies were counted. Each condition was repeated three times, on three independent cultures. Statistics were performed in Graphpad 6 software.

### Statistics

All data were analyzed in Graphpad Prism 6. To compare age at death between normal and ALS a Mann-Whitney Test was used. To compare disease duration between ALS disease cohorts a Kruskal-Wallis test was used. To compare cytoplasmic or nuclear PAR immunoreactivity between normal and ALS, or nuclear PAR between ALS and ALS-D cohorts a Fisher’s exact test was used. To compare nuclear PAR immunoreactivity between ALS-no mut, ALS-*ATXN2* and ALS-*c9*, a Chi square (*Χ*^2^) test was used. Cell culture and primary neuron experiments were repeated in triplicate and a mean (± SEM) is presented. One-way or two-way ANOVA followed by the appropriate post-hoc test was used to test for significance. Details of statistical tests used are in the associated figure legends. All experiments were repeated in triplicate unless otherwise stated. Data were considered statistically significant if *p* ≤ 0.05, *p* values are marked * if *p* ≤ 0.05, ** if *p* ≤ 0.01, *** if *p* ≤ 0.001 and **** if *p* ≤ 0.0001.

## Results

### Study subjects, clinical characteristics and diagnosis

**Fig. 1:**
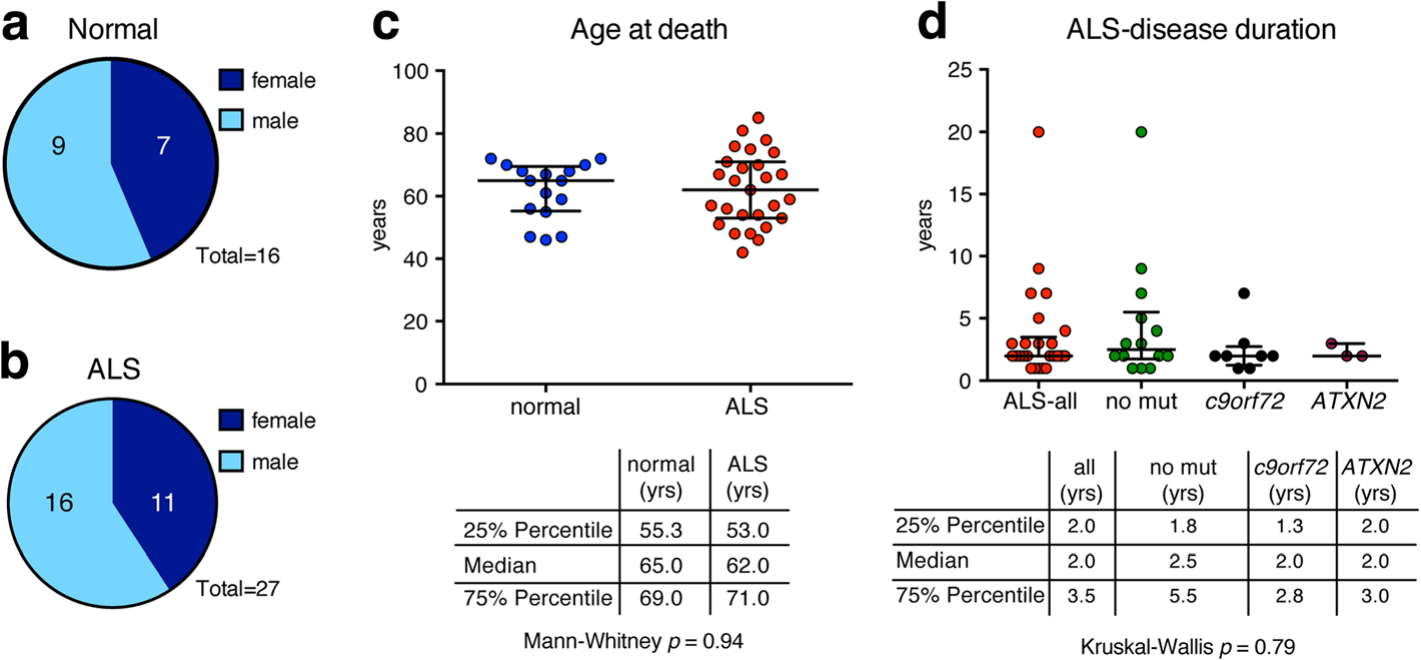
Case demographics. **a.** Spinal cord tissue from 16 patients with no history of neurodegenerative disease was examined in this study; 7 were female and 9 were male. **b.** The spinal cord from 27 patients diagnosed with ALS were examined in this study; 11 were female and 16 were male. **c.** There was no statistical difference in the age of death between the normal and ALS patients. The graph represents the median with interquartile range. A Mann-Whitney test was used to test for significance. **d.** Compared to the no-mutation carriers, the presence of a mutation in *C9orf72* or an intermediate polyQ expansion in *ATXN2* did not cause a significant change in disease duration in these pre-selected cohorts. The graph represents the median with interquartile range. A Kruskal-Wallis test was used to test for significance

We examined spinal cord tissue from a total of 43 patients; 16 were negative for any known neurological disorder (9 male and 7 female) and are described as the normal cohort (Fig. 1a and Table 1). As we wanted to analyze motor neurons and phosphorylated TDP-43 inclusions in the ALS spinal cord, we selected cases which had large alpha neurons that also contained phosphorylated TDP-43 inclusions. Of the 27 selected disease cases (16 male and 11 female), 17 were diagnosed with ALS, 8 with ALS concomitant with dementia (ALS-D), 1 with ALS concomitant with primary lateral sclerosis (PLS) and 1 with frontotemporal degeneration (FTD) (Fig. 1b and Table 2), and are collectively described as the ALS-cohort. The median age of onset for the disease cohort was 59 yr., the median disease duration was 2 yr. There was no significant difference in the median age at death between the normal and ALS cohorts (65 yr. vs 62 yr., respectively) (Fig. 1c). Of the ALS cohort, 14 were negative for known mutations in *TARDBP*, *UBQLN2*, *FUS, ATXN2* and *C9orf72,* 5 had an intermediate polyglutamine (polyQ) expansion (27–33 CAG repeats) in *ATXN2* (ALS-*ATXN2*)*,* and 8 cases had a G4C2-hexanucleotide repeat expansion in *C9orf72* (ALS-*c9*) (Table 2). No data for disease onset was present for two ALS-*ATXN2* cases. No significant difference was detected in this cohort for disease duration or age at death between ALS-no mut, ALS-*ATXN2* and ALS-*c9* (Fig. 1c-d).

### Nuclear PAR is elevated in motor neurons of ALS spinal cord

**Fig. 2:**
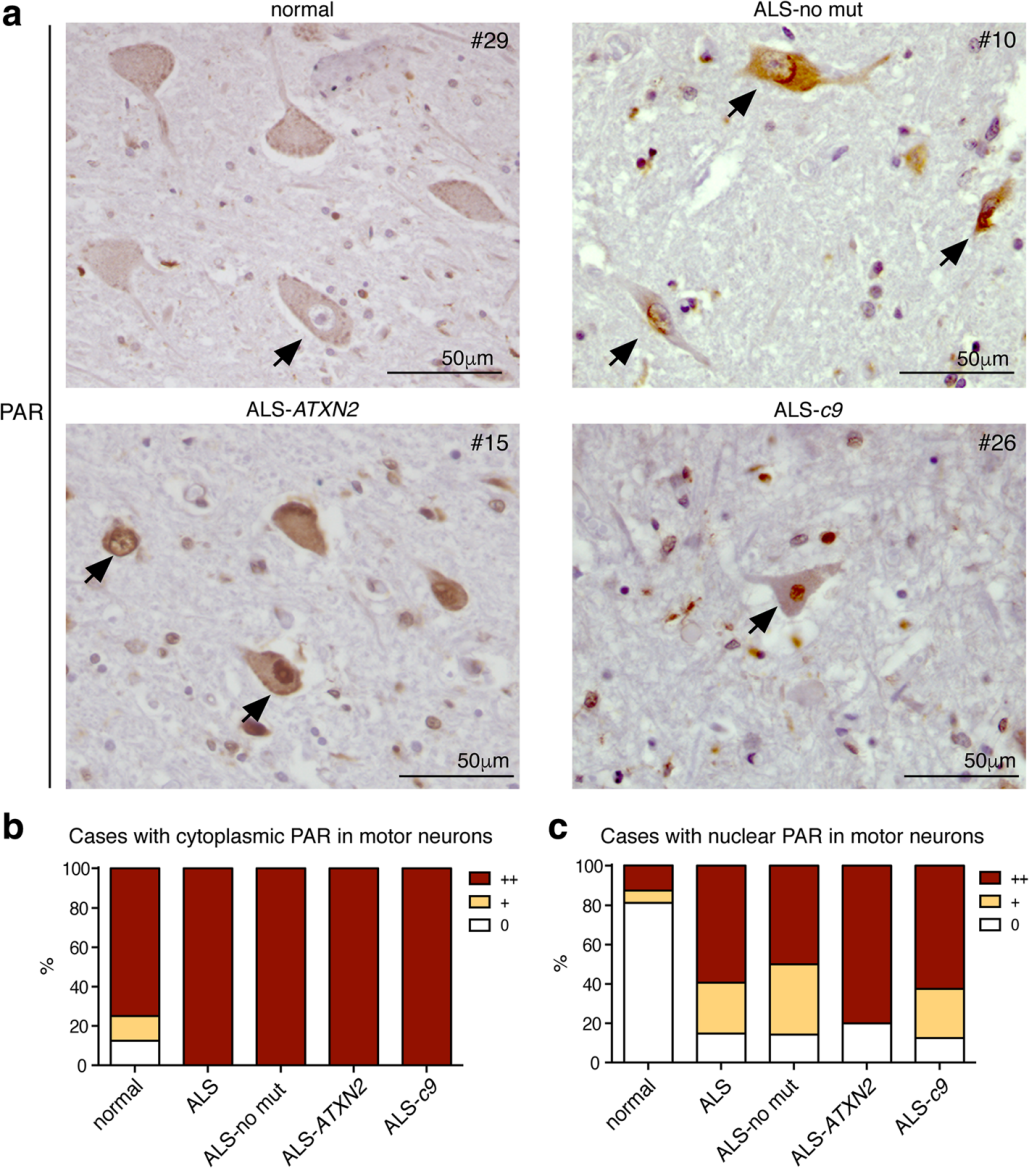
ALS motor neurons have elevated levels of nuclear PAR. **a**. Spinal cord sections from a neurologically normal case showing a motor neuron with no nuclear PAR immunoreactivity (arrow). An ALS-no mut case with three motor neurons with nuclear PAR (arrows). An ALS-*ATXN2* case with two motor neurons presenting with nuclear PAR (arrows). An ALS-*c9* case with one motor neuron with nuclear PAR (arrow). Sections were immunostained for PAR and counterstained with Hematoxylin. **b**. The presence of cytoplasmic PAR in the motor neurons of the spinal cord was quantified on a semi-quantitative scale (0 no detectable cytoplasmic PAR; + cytoplasmic PAR detected in 1 motor neuron; ++ cytoplasmic PAR detected in > 1 motor neuron), see also Tables 3 and 4. The data was charted as a percentage. **c**. The presence of nuclear PAR in the motor neurons of the spinal cord was quantified on a semi-quantitative scale (0 no detectable nuclear PAR; + nuclear PAR detected in 1 motor neuron; ++ nuclear PAR detected in > 1 motor neuron), see also Tables 3 and 4. The data were charted as a percentage. Slides were fully blinded and examined independently by two researchers, images for figures were captured with a 20X objective and an optivar magnification of 1.6

To ascertain whether PAR activity was misregulated in disease, we examined the post-mortem spinal cord for immunoreactivity against PAR. We observed PAR in the nucleus and cytoplasm of motor neurons in spinal-cord tissue from both neurologically normal and ALS patients (Fig. 2a). Tissue sections were coded and blinded and examined for the presence of PAR in the motor neurons of the anterior horn. The severity of neuropathological markers such as phosphorylated TDP-43 are routinely graded on a semi-quantitative scale [14, 15]. We developed a semi-quantitative scale to score PAR immunoreactivity in motor neurons (0 not detectable; + detectable in 1 motor neuron; and ++ detectable in > 1 motor neuron) and examined staining in both the cytoplasm and nucleus. Our analysis revealed that 12 out of 14 of the neurologically normal cases and 27 out of 27 ALS cases presented with PAR in the cytoplasm of motor neurons (Fig. 2a-b and Tables 3 and 4). A Fisher’s exact test revealed no significant difference (*p* = 0.1329) between normal and ALS patients, indicating that cytoplasmic PAR was not significantly misregulated in this disease cohort. By contrast, nuclear PAR in the spinal cord motor neurons was detected in 3 out of 16 normal cases and in 24 out of 27 ALS cases (Fig. 2a and c, Tables 3 and 4). All cases that were negative for nuclear PAR presented with motor neurons with visible nuclei. A Fisher’s exact test between the normal and ALS cases revealed that motor neurons with nuclear PAR was significantly (*p* < 0.0001) associated with ALS. Additionally, the presence of nuclear PAR in the motor neurons of the spinal cord from ALS-no mut, ALS-*ATXN2* and ALS-*c9orf72* did not differ (*Χ*^2^ (3) = 0.1436, *p* = 0.9861) (Table 4). Given the reported morphological differences in TDP-43 aggregates in the anterior cingulate of ALS vs ALS-D patients [106], we compared nuclear PAR in the motor neurons between these two disease subtypes and observed no statistical significance (*p* = 1.0). It is important to note that the normal anterior horn compared to the ALS-all anterior horn had significantly more motor neurons (19.5±1.4 vs 13.7±1.4 (SEM) *p* = 0.0081) and significantly more visible nuclei (5.6±0.6 vs 4.0±0.4 (SEM) *p* = 0.0393). It is likely that the severity of nuclear PAR staining in ALS is under represented in our analyses. Combined, our data indicate that the motor neurons of the post-mortem spinal cord from ALS patients exhibit significantly elevated levels of nuclear PAR.

**Table 3:** PAR immunoreactivity in patients with no known neurological disease

#	Diagnosis	Region analyzed	PAR in MN nuclei	PAR in MN cytoplasm
1	normal	cervical	0	0
2	normal	cervical	0	++
3	normal	cervical	++	++
4	normal	cervical	0	++
5	normal	cervical	0	0
6	normal	cervical	0	++
7	normal	lumbar	+	+
8	normal	cervical	0	+
9	normal	lumbar	0	++
10	normal	cervical	0	++
11	normal	cervical	0	++
12	normal	cervical	0	++
13	normal	cervical	++	++
14	normal	thoracic	0	++
15	normal	cervical	0	++
16	normal	cervical	0	++

Abbreviations: #: case number. *Normal* diagnosed neurologically normal. *F* female, M, male. *PAR* poly(ADP-ribose). *MN* motor neuron

**Table 4:** PAR immunoreactivity in patients diagnosed with neurological disease

#	Diagnosis	Mutation Status	Region analyzed	PAR in MN nuclei	PAR in MN cytoplasm
17	ALS	–	cervical	+	++
18	ALS	–	lumbar	++	++
19	ALS	–	cervical	++	++
20	ALS-D	–	lumbar	++	++
21	ALS	–	lumbar	++	++
22	ALS	–	lumbar	++	++
23	ALS	–	lumbar	0	++
24	ALS	–	cervical	+	++
25	ALS	–	cervical	+	++
26	ALS-D	–	cervical	++	++
27	ALS/PLS	–	cervical	+	++
28	ALS	–	cervical	0	++
29	ALS	–	lumbar	++	++
30	ALS	–	cervical	+	++
31	ALS	*ATXN2 (22/32)*	cervical	++	++
32	ALS-D	*ATXN2 (22/27)*	cervical	++	++
33	ALS	*ATXN2 (20/31)*	thoracic	++	++
34	ALS	*ATXN2 (22/29)*	lumbar	++	++
35	ALS	*ATXN2 (*22/27)	cervical	0	++
36	ALS	*C9orf72*	cervical	++	++
37	FTD	*C9orf72*	cervical	++	++
38	ALS-D	*C9orf72*	cervical	++	++
39	ALS-D	*C9orf72*	thoracic	0	++
40	ALS-D	*C9orf72*	thoracic	++	++
41	ALS-D	*C9orf72*	cervical	+	++
42	ALS-D	*C9orf72*	lumbar	++	++
43	ALS	*C9orf72*	sacral	+	++

Abbreviations: #: case number. -: No known mutation in *TARDBP*, *UBQLN2*, *ATXN2*, and *C9orf72*. *ATXN2* refers to an intermediate CAG-trinucleotide expansion in *ATXN2* (pathologic repeat length is indicated in brackets). *C9orf72* refers to a GGGGCC-hexanucleotide repeat expansion. *ALS-D* ALS with dementia, *FTD* frontotemporal degeneration, *PLS* primary lateral sclerosis, *PAR* poly(ADP-ribose), *MN* motor neuron

### Motor neurons do not have cytoplasmic inclusions of PAR

**Fig. 3:**
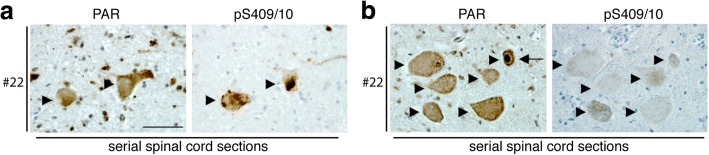
PAR does not form protein aggregates in the cytoplasm of motor neurons. **a.** Serial sections from ALS spinal cord tissue were stained for either PAR or phosphorylated TDP-43 (pS409/10). Motor neurons with phosphorylated TDP-43 aggregates did not also have cytoplasmic aggregates labelled with PAR. Arrowheads indicate the same neurons in each serial section. Scale bar: 50 μm. **b.** Serial sections of ALS spinal cord tissue were stained for either PAR or phosphorylated TDP-43 (pS409/10). The motor neurons shown with elevated nuclear PAR did not have cytoplasmic aggregates of phosphorylated TDP-43. Arrowheads indicate the same neuron in each section. Arrows indicate neuron with nuclear PAR. Scale bar: 50 μm

To gain further insight into the pattern of PAR immunoreactivity in the ALS spinal cord, we determined whether PAR formed neuronal cytoplasmic inclusions in neurons that contained phosphorylated TDP-43. Serial sections of spinal cord from 4 ALS patients were immunostained with either an antibody that selectively detects TDP-43 phosphorylated at serines 409/410 (pS409/10) or with an antibody that detects PAR. In all 4 cases (case numbers: 22, 23, 25 and 26) cytoplasmic inclusions of phosphorylated TDP-43 were present in the motor neurons (Fig. 3a). In serial sections, we found no evidence of PAR aggregation in the cytoplasm in the neurons in which phosphorylated TDP-43 was detected (Fig. 3a). Additionally, in these 4 cases (22, 23, 25 and 26) nuclear PAR was present in motor neurons, and in serial sections none of those motor neurons displayed phosphorylated TDP-43 pathology (Fig. 3b). These data indicate that nuclear PAR occurred in motor neurons that have not developed phosphorylated TDP-43 pathology.

### Veliparib suppresses the formation of stress-induced foci of TDP-43

Since nuclear PAR was detected in motor neurons of the ALS spinal cord, the activity of the nuclear PARPs may be activated in disease. There are three nuclear PARP enzymes: PARP-1, PARP-2, and also PARP-3, which is a mono(ADP-ribose) transferase [41, 48, 60]. The antibody used to detect PAR recognizes PAR chains of 20 or more ADP-ribose subunits [52], suggesting that the PAR detected in the ALS spinal cord (see Figs. 2 and 3) is generated from PARP-1 or PARP-2 (collectively known as PARP-1/2). Small-molecule inhibitors of PARP-1/2 activity have been pursued as cancer therapeutics in more than 300 FDA-approved clinical trials [54, 102]. We thus sought to determine if PARP-1/2 inhibition could be of potential therapeutic value to ALS.

**Fig. 4:**
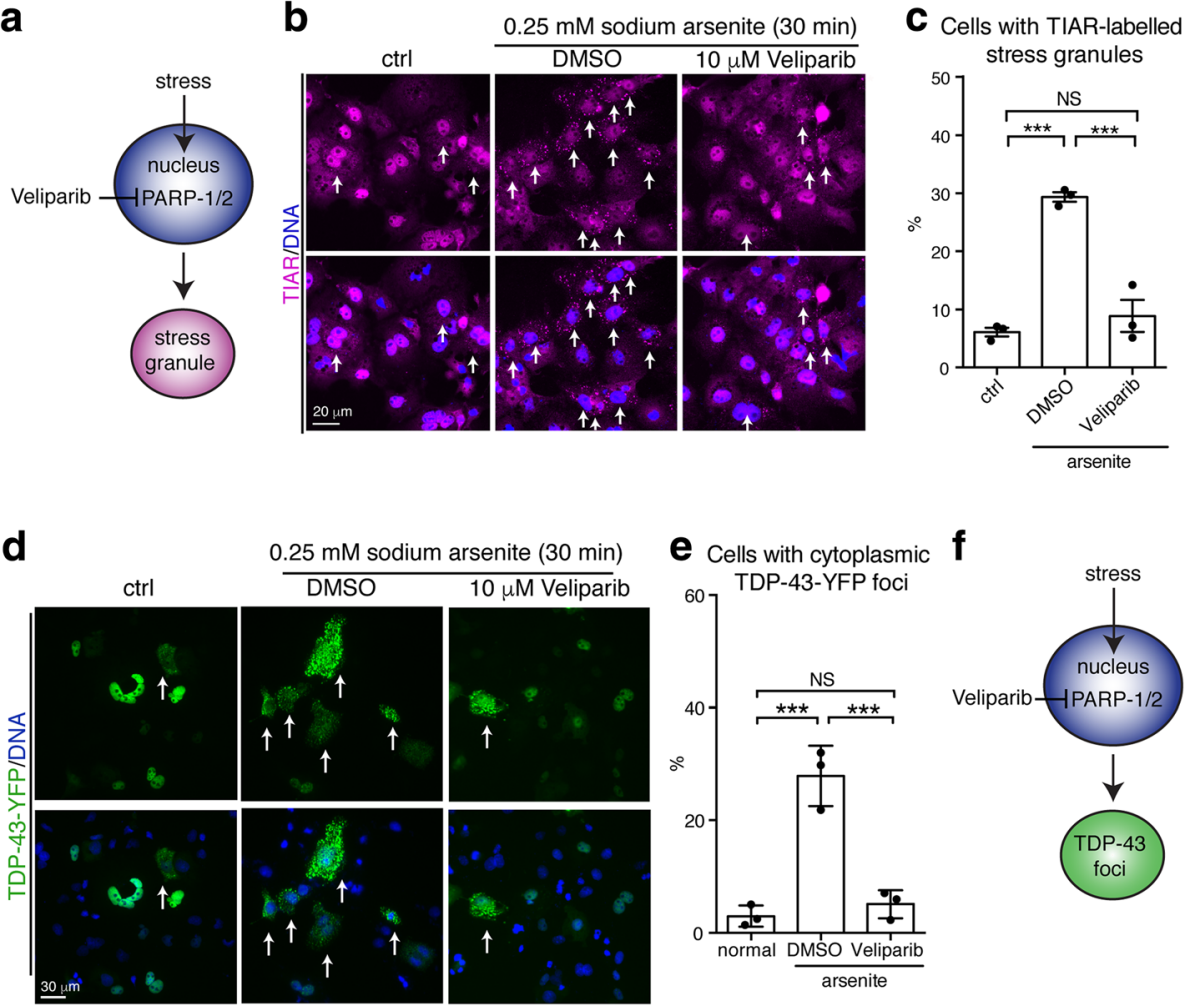
Small molecule inhibition of PARP-1/2 reduces the formation of stress-induced TDP-43 foci in mammalian cells. **a** Veliparib is a small molecule inhibitor of PARP-1/2 activity reported to inhibit the formation of G3BP1-labelled foci in the cytoplasm upon UV treatment [49]. **b** Exposure to arsenite leads to the formation of TIAR-labelled stress granules in the cytoplasm (arrows). Co-treatment with Veliparib inhibits the formation of TIAR-labelled stress granules. COS-7 cells transfected with TDP-43-YFP were immunostained for TIAR and counterstained with Hoescht. Cells were imaged for TIAR and Hoescht. **c** Cells were quantified for the presence of cytoplasmic TIAR-labelled stress granules. Mean (± SEM) is presented. One-way ANOVA followed by a Tukey’s test was used for significance. **d** Under normal conditions (ctrl), TDP-43-YFP diffusely localizes to the nucleus of COS-7 cells. Upon treatment with arsenite, TDP-43-YFP forms foci in the cytoplasm (arrows). The formation of cytoplasmic TDP-43-YFP foci is inhibited by treatment with Veliparib. Cells were counterstained with Hoescht. **e** Veliparib reduces the accumulation of TDP-43-YFP foci in the cytoplasm. Cells were quantified for the presence of cytoplasmic TDP-43-YFP foci. Mean (± SEM) is presented. One-way ANOVA (*p* = 0.0002) followed by a Tukey’s test was used for significance. **f** Hypothetical schematic showing that inhibition of PARP-1/2 activation by Veliparib inhibits the formation of stress-induced TDP-43-YFP foci

In cells, TDP-43 can be induced to aggregate and localize to cytoplasmic stress granules. It has been reported that the PARP-1/2 inhibitor, Veliparib, inhibits the formation cytoplasmic stress granules [23, 49]. We determined the efficacy of Veliparib to mitigate the formation of arsenite-induced TIAR-labelled stress granules in COS-7 cells (Fig. 4a). Upon exposure to 0.25 mM sodium arsenite, the percentage of cells with TIAR-labelled stress granules increased from 6±1% to 29±2% (SEM) (Fig. 4b-c). Co-treatment with 10 μM Veliparib reduced the percentage of cells with arsenite-induced TIAR-labelled stress granules to 9±1% (SEM) (Fig. 4b-c). To examine the efficacy of Veliparib to mitigate cytoplasmic aggregation of TDP-43 in COS-7 cells, we exogenously expressed TDP-43-YFP. Normally, TDP-43-YFP was diffusely nuclear, however upon exposure to 0.25 mM sodium arsenite the percentage of cells with cytoplasmic TDP-43-YFP foci increased from 3±1% to 30±1% (SEM) (Fig. 4d-e). Co-treatment with Veliparib significantly reduced the percentage of cells with arsenite-induced TDP-43-YFP foci to near control levels (5±1% (SEM)) (Fig. 4d-e). These data suggest that in response to arsenite exposure, PARP-1/2 activity regulates stress-granule formation and stress-induced TDP-43 aggregation in the cytoplasm.

### Veliparib mitigates TDP-43 toxicity in primary spinal cord neurons

**Fig. 5:**
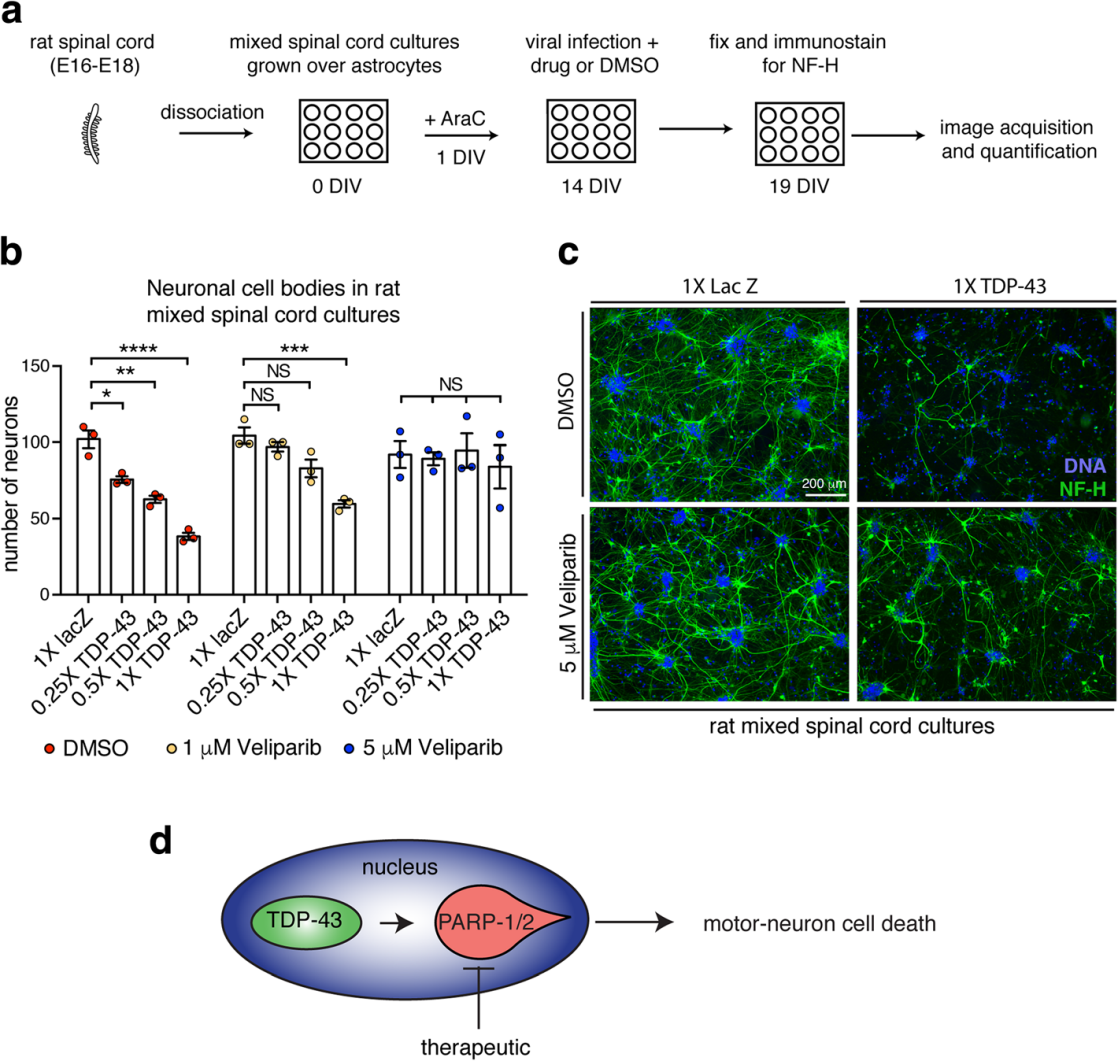
Veliparib inhibits TDP-43-associated neuronal loss in rat spinal cord cultures. **a.** The spinal cord was isolated from Sprague Dawley embryos (E16-E18), dissociated with protease and DNase, and seeded onto astrocyte coated 12-well plates. After 1 day in vitro (1 DIV) cell proliferation was stopped by the addition of 5 μM cytosine arabinoside (AraC). At 14 DIV cultures were infected with a LacZ control or TDP-43 attenuated herpes simplex virus alongside DMSO or Veliparib. At 19 DIV the neurons were fixed and immunostained for the neuronal marker neurofilament-H (NF-H) and counterstained with Hoescht. Five images (10X magnification) were captured from each condition and neuronal cell bodies were counted. Each condition was repeated three times from 3 independent cultures. **b**. Viral infection of TDP-43 leads to the loss of neuronal cell bodies in dose-dependent manner. Co-treatment with 1 μM or 5 μM Veliparib inhibits TDP-43-induced neuronal cell loss. Mean (± SEM) is presented, each data point represents three technical repeats from an independent culture. 1X represents a virus titer of 3-5 × 10^4^ pfu/ml. Two-way ANOVA (*p* < 0.0001) and a Dunnett’s test for significance was performed. NS: not significant. **c.** Example images (magnification 10X), of rat spinal-cord cultures infected with 1X LacZ or 1X TDP-43 and incubated with DMSO or 5 μM Veliparib. Cultures were immunolabeled for Neuro filament-H (NF-H) and counterstained with Hoescht. **d**. Schematic showing that motor neuron loss induced by virally expressed TDP-43 in spinal cord cultures and that this loss is suppressed by the PARP-1/2 inhibitor Veliparib

Since Veliparib inhibited the accumulation of stress-induced TDP-43 foci in the cytoplasm, we queried whether this treatment could impact the toxicity of TDP-43 to primary spinal cord cultures. To address this question, we developed a toxicity assay in mixed spinal-cord cultures isolated from rat embryos (Fig. 5a). The primary spinal cord cultures were virally infected with an attenuated herpes simplex virus expressing a LacZ control or of TDP-43. The cultures were maintained for 5d post infection, after which they were immunostained for the neuronal specific marker Neurofilament-H (NF-H) and the remaining NF-H-labeled cell bodies were imaged and quantified. In control conditions (LacZ), we observed an average of 102±5.6 (SEM) neuronal cell bodies (Fig. 5b-c). Infection with TDP-43 at 0.25×, 0.5× and 1× resulted in a dose-sensitive loss of neurons (76±2.2, 63±2.4 and 38±2.4 (SEM) neuronal cell bodies respectively) (Fig. 5b-c), indicating that virally expressed TDP-43 results in neuronal cell loss in rat spinal cord cultures.

To determine if Veliparib was effective at mitigating TDP-43-associated neuronal degeneration, we first examined spinal cord cultures infected with the LacZ control and treated with either DMSO or with 1 μM or 5 μM Veliparib. These controls revealed that Veliparib had no deleterious effects on the mixed spinal cord cultures at the concentrations tested (Fig. 5b-c). We then compared rat spinal cord cultures infected with TDP-43 and co-treated with DMSO or 1 μM or 5 μM Veliparib. Notably, treatment with 5 μM Veliparib protected the primary neurons such that there was no significant difference in the number of neuronal cell bodies, at all infection ratios of TDP-43 compared to the DMSO control (Fig. 5b-c). The neuronal processes appeared retained although not to the level of the control (Fig. 5c). These studies cannot determine whether the neurons or astrocytes account for TDP-43-associated neuronal loss or for the beneficial action of Veliparib. However, small molecule inhibition of PARP-1/2 is effective in mitigating TDP-43-associated neuronal loss in these spinal cord cultures, and could have therapeutic utility for ALS and other TDP-43-associated diseases.

## Discussion

Our data indicate that ALS is associated with elevated nuclear PAR in the motor neurons of the spinal cord in all genetic backgrounds tested (no mutation, intermediate polyQ expansion in *ATXN2* or *C9orf72* mutation). We show that Veliparib, an inhibitor of nuclear PARP-1/2 activity, mitigates the formation of stress-induced cytoplasmic aggregates of TDP-43 in mammalian cells. We extend this finding to show that treating rodent spinal-cord cultures with Veliparib mitigates TDP-43-induced neuronal cell loss. Collectively, our data implicate the misregulation of nuclear PARP activity in ALS and highlight PARP-1/2 inhibitors as potential compounds for further therapeutic research.

In the early stages of ALS some patients will present with symptoms of neuronal hyperexcitability such as fasciculation and cramp [7, 111]. In support of hyperexcitability as a physiological mechanism, glutamate, the major excitatory neurotransmitter in the CNS, is elevated in the cerebrospinal fluid of ALS patients [93, 94, 101, 103]. Notably, PARP-1 activation has been implicated in meditating the response to glutamate-induced neurotoxicity in animal and cellular assays [5, 24, 113, 116]. Our neuropathologic analyses demonstrate that long-chained PAR polymers are present at elevated levels in the motor-neuron nuclei of the ALS spinal cord. This finding implicates activation of the nuclear PARP enzymes (PARP-1 and PARP-2). PARP-1 is the most abundant and the most active following stress [27, 67]. The downstream consequence of PARP-1 activation leads to the propagation of several stress-associated pathways [1, 21, 34, 63]. Upon over activation of PARP-1, the enzyme elicits a cell death mechanism, which is characterized by cleavage of PARP-1 [34, 50, 100]. Previous reports indicate that PARP-1 protein and cleaved PARP-1 is elevated in ALS [32, 56, 57]. Combined with our data that demonstrate that PAR is elevated in ALS motor neurons it could be that PARP-1/2 is activated by localized glutamate excitotoxicity and that the motor neurons may be undergoing PAR-mediated cell death.

PARP-1/2 also plays a role in nuclear and cytoplasmic protein localization. For example, upon inflammatory stress, PARP-1/2 promotes nuclear retention of the transcription factor High mobility group B1 (HMGBP1) [1]. Under extreme conditions of DNA damage PAR polymers produced by PARP-1 are released into the cytoplasm and bind to Apoptosis Inducing Factor (AIF) in the mitochondria to promote translocation of AIF and macrophage migration inhibitory factor (MIF) to the nucleus to elicit a programmed cell death mechanism [34, 113, 114]. PARP-1 activity has also been implicated in signaling to PARP-12 in the cytoplasm to regulate the formation of cytoplasmic stress granules [23, 49]. Here we show that treatment with PARP-1/2 inhibitor, Veliparib, mitigates the formation of stress-induced aggregates of TDP-43 in the cytoplasm, suggesting that PARP-1/2 activity impacts cytoplasmic aggregation of TDP-43. Indeed, nuclear PAR was not detected in neurons harboring phosphorylated TDP-43 aggregates, suggesting that the PARP-1/2 activation observed in ALS motor neurons may occur at earlier stages in neuron compromise. We suggest that PARP-1/2 activation may precede the exit of TDP-43 from the nucleus and the subsequent formation of cytoplasmic TDP-43 aggregates.

Of the ~ 5% of ALS cases that lack TDP-43 pathology (TDP-43-negative ALS), a subset is the result of a mutation in *FUS (*fused in sarcoma) [46, 61, 112]. FUS is an RNA-binding protein that is recruited to sites of DNA damage by PARP-1 [3, 81, 96] and at high concentrations, the PARP-1/2 inhibitor Veliparib can promote the mislocalization of nuclear FUS-GFP to the cytoplasm [81]. A second notable gene mutated in TDP-43-negative ALS is *SOD1* (superoxide dismutase 1) [69]. Curiously, PARP-1 protein is elevated in spinal cord astrocytes in SOD1 G93A transgenic mice [22] and is cleaved in SOD-1 cellular models [59]. However, pharmacological treatment with a PARP-1 inhibitor had no effect on the lifespan or motor performance of the SOD1 G93A transgenic mouse [6]. It is possible that PARP-1/2 regulation of neuronal demise is selectively involved in TDP-43-positive ALS. In support of PARP-1 mediated regulation of the central nervous system in disease it has been shown that PARP-1 overactivation leads to neuronal degeneration in *Drosophila* [43]. PARP-1 activation has also been linked to Alzheimer’s (AD), Parkinson’s (PD) and ischemic stroke [19, 31, 51, 71, 72, 79], and the use of PARP-1/2 inhibitors is beneficial to mouse models of these diseases [2, 20, 25, 30, 87, 105, 109, 115]. These data indicate that, despite dampening the DNA damage response, PARP-1/2 inhibition provides improved neuronal integrity and function in these animal models of disease. To assign therapeutic potential of PARP-1/2 inhibitors and understand potential side effects, it will be imperative to examine additional ALS subtypes and associated diseases.

A range of small-molecule inhibitors of PARP-1/2, including the inhibitor used here, have been developed for clinical application as they sensitize cancer cells to cell death. Moreover, some have been reported to cross the blood-brain barrier [16, 17, 33]. These inhibitors have been tested in hundreds of FDA-approved clinical trials of various cancers and there is a wealth of information on the pharmacokinetics, pharmacodynamics, and toxicity of these compounds that would be beneficial in repurposing them for alternative diseases [11, 54, 102]. We previously implicated inhibitors of PARP-5a and PARP-5b, collectively known as PARP-5a/5b, in reducing the cytoplasmic aggregation of TDP-43, without having an effect on the percentage of cells with G3BP1-positive stress granules [73]. PARP-5a/5b inhibitors are also in development as cancer therapeutics [42, 62, 70]. It is possible that PARP-1/2 more broadly effects stress granule formation and stress-induced protein aggregation, while PARP-5a/5b may act on select proteins in stress signaling.

## Conclusion

Our study implicates the activation of PARP-1/2 in the motor neuron nuclei of the ALS spinal cord. We show that treatment with Veliparib, a PARP-1/2 inhibitor, reduces stress-induced accumulation of TDP-43 in the cytoplasm of mammalian cells. Furthermore, we show that Veliparib can mitigate the toxic effect of virally expressed TDP-43 in rodent spinal cord cultures. Currently, the mechanisms that may link TDP-43 and PARP-1/2 in cell culture models and human disease remains to be elucidated. We suggest that the PARP superfamily is an area that should be explored further in ALS therapeutics.
